# Mitotic CDK1 and 4E-BP1 I: Loss of 4E-BP1 serine 82 phosphorylation promotes proliferative polycystic disease and lymphoma in aged or sublethally irradiated mice

**DOI:** 10.1371/journal.pone.0282722

**Published:** 2023-05-05

**Authors:** Rui Sun, Siying Guo, Yoko Shuda, Anish B. Chakka, Lora H. Rigatti, Guangyi Zhao, Mohammed A. E. Ali, Christopher Y. Park, Uma Chandran, Jian Yu, Christopher J. Bakkenist, Masahiro Shuda, Patrick S. Moore, Yuan Chang

**Affiliations:** 1 Cancer Virology Program, UPMC Hillman Cancer Center, Pittsburgh, Pennsylvania, United States of America; 2 Department of Biomedical Informatics, University of Pittsburgh, Pittsburgh, Pennsylvania, United States of America; 3 Division of Laboratory Animal Resources, University of Pittsburgh Cancer Institute, Pittsburgh, Pennsylvania, United States of America; 4 Department of Pathology, University of Pittsburgh School of Medicine, UPMC Hillman Cancer Center, Pittsburgh, Pennsylvania, United States of America; 5 Department of Pathology, NYU Grossman School of Medicine, Perlmutter Cancer Center, New York, New York, United States of America; 6 Radiation Oncology, UPMC Hillman Cancer Center, Pittsburgh, Pennsylvania, United States of America; Ohio State University, UNITED STATES

## Abstract

4E-BP1 is a tumor suppressor regulating cap-dependent translation that is in turn controlled by mechanistic target of rapamycin (mTOR) or cyclin-dependent kinase 1 (CDK1) phosphorylation. 4E-BP1 serine 82 (S82) is phosphorylated by CDK1, but not mTOR, and the consequences of this mitosis-specific phosphorylation are unknown. Knock-in mice were generated with a single 4E-BP1 S82 alanine (S82A) substitution leaving other phosphorylation sites intact. S82A mice were fertile and exhibited no gross developmental or behavioral abnormalities, but the homozygotes developed diffuse and severe polycystic liver and kidney disease with aging, and lymphoid malignancies after irradiation. Sublethal irradiation caused immature T-cell lymphoma only in S82A mice while S82A homozygous mice have normal T-cell hematopoiesis before irradiation. Whole genome sequencing identified PTEN mutations in S82A lymphoma and impaired PTEN expression was verified in S82A lymphomas derived cell lines. Our study suggests that the absence of 4E-BP1^S82^ phosphorylation, a subtle change in 4E-BP1 phosphorylation, might predispose to polycystic proliferative disease and lymphoma under certain stressful circumstances, such as aging and irradiation.

## Introduction

4E-binding protein 1 (4E-BP1) is a regulator of protein synthesis and has been implicated as a tumor suppressor [1] in a variety of neoplasias including Ewing sarcoma [2], head and neck cancer [3], and colon cancer [4]. 4E-BP1 regulates cap-dependent translation of specific classes of mRNAs, particularly those encoding 5’-terminal oligopyrimidine (5’-TOP) sequences [5]. In its unphosphorylated state, 4E-BP1 is active, and binds to eIF4E, thereby preventing assembly of the eIF4F protein complex on the mRNA 5’ cap required for ribosomal initiation of mRNA translation [6]. Multi-phosphorylation of 4E-BP1 by mTOR complex 1 (mTORC1) kinase inactivates 4E-BP1, releasing eIF4E and allowing translation initiation for specific mRNA transcripts [7].

The small T oncoprotein of Merkel cell polyomavirus, or MCV, which causes Merkel cell carcinoma [8], is a potent transforming oncoprotein in rodent fibroblast transforming assays [9]. MCV sT induces mitotic arrest [9] by targeting several E3 ligases, including Fbw7 [10] and the anaphase promoting complex/cyclosome (APC/C) [11]. An intact sT E3 ligase binding domain (called the Large T stabilizing domain or LSD/LTSD) is required for this cell transformation [10]. MCV small T expression causes metaphase arrest and induction of active cyclin-dependent kinase 1 (CDK1 or cdc2)/cyclin B [12], which can substitute for mTORC1 to hyperphosphorylate 4E-BP1 during mitosis [13]. This mTORC1-to-CDK1 mitotic switch allows CDK1 to inactivate mTORC1 by phosphorylating raptor protein [14], but at the same time supplants the function of mTORC1 to phosphorylate some mTORC1 substrates during mitosis [15–17].

The 4E-BP1 amino acid residues phosphorylated by both mTORC1 and CDK1/cyclin B include threonine (T)36/T47, serine (S)65 and T70 [13,18]. Substitution of these residues with alanine (A), particularly the priming T36 and T47 residues [19], results in a constitutively active 4E-BP1 [20]. In addition to all of these sites, CDK1 also phosphorylates 4E-BP1 residue S82 in mice (S83 in humans) during mitosis. This site is not phosphorylated by mTOR during interphase [13,18]. The function for mitotic S82 phosphorylation is unknown, but expression of human 4E-BP1 with alanine substitution at S83 in trans reduces the efficiency of MCV sT-induced rodent cell transformation [21].

To study the function of 4E-BP1^S82^ phosphorylation, we established homozygous S82 alanine substitution (S82A) knock-in C57/B6N mice. Aged homozygous S82A mice developed degenerative polycystic kidney and liver disease not present in wild-type littermates. On 9 Gy total body irradiation, S82A mice developed T cell lymphomas that were not present in irradiated wild-type littermates. These phenotypes are remarkable given that other phosphorylation sites in 4E-BP1 were functionally intact [18] and mice in which the entire *Eif4ebp1* gene is deleted remain viable [22]. Our study suggests that the absence of 4E-BP1^S82^ phosphorylation by CDK1, effected by a single amino acid substitution from S82 to alanine, can predispose to polycystic proliferative disease and lymphoma.

## Results

### Aged S82A mice are prone to polycystic liver and kidney disease and lymphoma

4E-BP1^S82A^ knock-in mice were generated using a Cre-loxP cloning strategy (S1A Fig). A hemizygous founder was used to generate heterozygous 4E-BP1^S82A^ mice, which in turn were used to generate the homozygous *Eif4ebp1*^S82A/S82A^ (**S82A**) and *Eif4ebp1*^WT/WT^ (**WT**) littermate mice used in this study. The generation of a single point mutation was confirmed by genotyping PCR, immunoblotting and whole genome sequencing (WGS) (S1B–S1D Fig). Littermate S82A and WT mice were fertile and exhibited no gross developmental or behavioral abnormalities.

**Fig 1:**
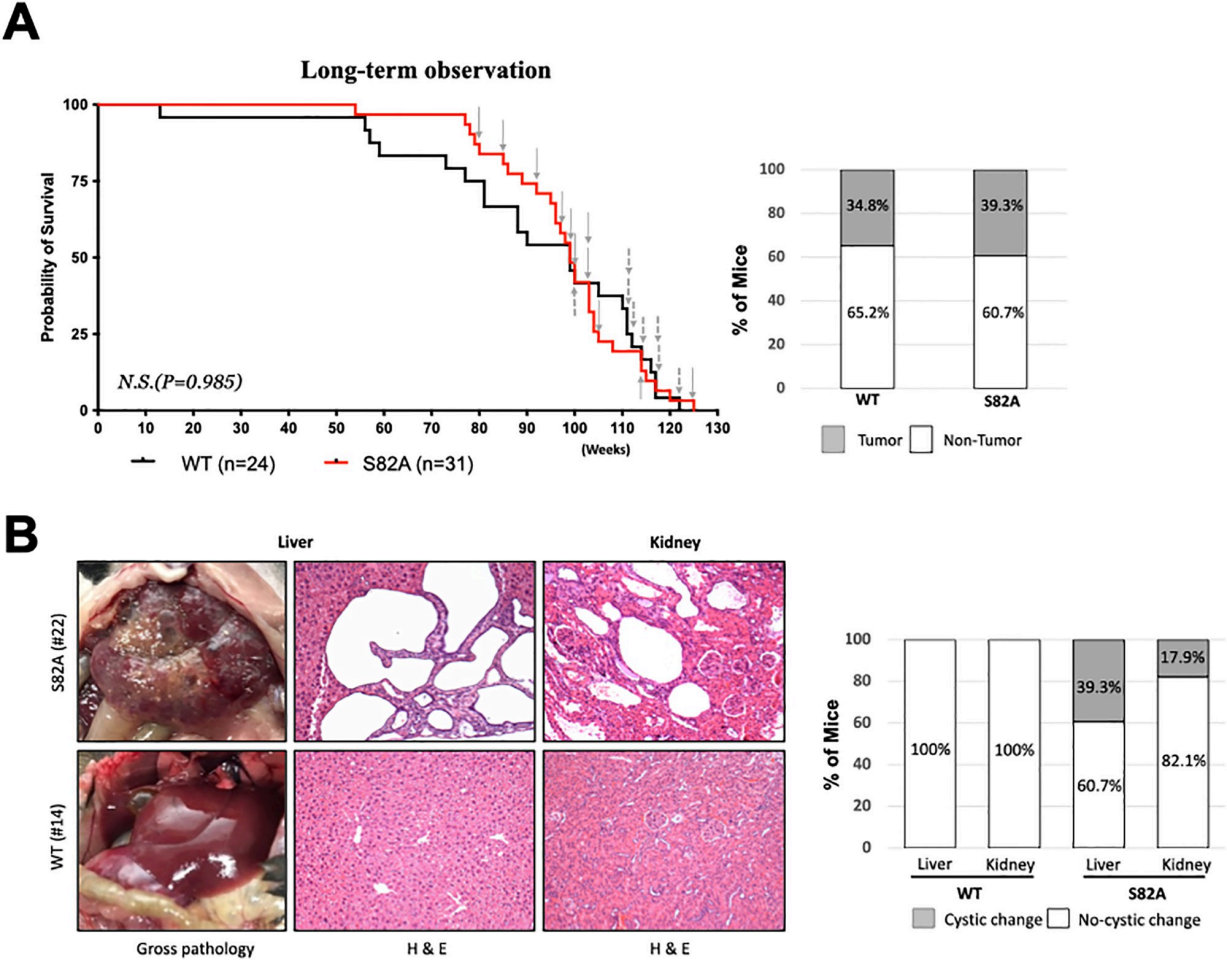
Homozygous 4E-BP1^S82A^ mice develop lymphomas and polycystic liver and kidney disease during aging. **(A)** Kaplan–Meier survival curve (left panel). S82A mice (n = 31, red line) and WT mice (n = 24, black line) were subjected to long-term observation. Mice with 20% weight loss or illness were euthanized and processed for gross anatomic and histologic examination. Log-rank (Mantel-Cox) test was used to compare survival probability. N.S. indicates difference is not significant. 11 of 28 (39.3%) S82A mice and 8 of 23 (34.8%) WT mice died with observable lesions. Chronologic occurrence of lesions are marked with grey arrows (solid for S82A and dashed for WT). Column plot (right panel) summarizes the percentage of mice with tumors (grey shading) found in S82A and WT and mice. **(B)** S82A mice aging mice develop polycystic liver and kidney lesions. On gross examination, S82A livers in some mice were enlarged by variably-sized cysts containing clear to yellow fluid affecting all lobes. These areas correspond microscopically to loss of hepatic parenchyma and replacement by cystic spaces lined by cuboidal epithelium within a fibrous connective tissue stroma containing lymphocytic infiltrates. S82A kidneys with cystic lesions microscopically had elongated cysts of varying sizes within the renal cortex. Cysts were lined by cuboidal to attenuated epithelial cells (Top row). Cystic lesions were not found in WT animals (Bottom row).

Overall survival did not significantly differ between S82A and WT mice (Fig 1A). Tumor masses were detected in eleven of twenty-eight (39.3%) S82A mice and in eight of twenty-three (34.8%) WT mice (Fig 1B). Eight of twenty-eight (28.6%) S82A mice developed lymphocytic lymphomas compared to four of twenty-three (17.4%, p = 0.51 FET) WT mice (Table 1 and Fig 1A). Seven of the eight S82A lymphomas were of CD19+ B cell origin, as were all four of the WT lymphomas. Other sporadic cancers, including angiosarcoma and adenocarcinoma, did not significantly differ between the two mouse strains (Table 1). No splenomegaly or lymphadenopathy was detectable on gross examination in the S82A mouse strain.

**Table 1:** Long-term observation on WT and S82A mice.

Group	Mouse #	Age at death	Tumor	Cystic change in Liver	Cystic change in Kidney
WT(n = 24)	1	13w	N.A.	N.A.	N.A.
	2	56w 5d	-	-	-
	3	57w 4d	-	-	-
	4	59w	-	-	-
	5	73w	-	-	-
	6	77w	-	-	-
	7	81w 6d	-	-	-
	8	81w6d	-	-	-
	9	88w4d	-	-	-
	10	88w2d	-	-	-
	11	90w	-	-	-
	12	99w	-	-	-
	13	99w2d	-	-	-
	14	100w1d	**B-cell Lymphoma**	-	-
	15	105w5d	-	-	-
	16	110w	-	-	-
	17	111w2d	**Histiocytic sarcoma**	-	-
	18	111w5d	Adenocarcinoma	-	-
	19	112w	Angiosarcoma	-	-
	20	114w2d	**B-cell Lymphoma**	-	-
	21	116w5d	-	-	-
	22	117w2d	**B-cell Lymphoma**	-	-
	23	117w6d	**Histiocytic sarcoma**	-	-
	24	122w3d	**B-cell Lymphoma**	-	-
S82A (n = 31)	1	54w2d	-	++	-
	2	77w6d	-	-	-
	3	78w3d	-	+++	-
	4	79w6d	**T-cell Lymphoma**	-	-
	5	80w3d	-	-	-
	6	85w	**B-cell Lymphoma**	-	-
	7	86w3d	-	-	-
	8	89w4d	-	-	-
	9	92W	**Histiocytic sarcoma**	-	-
	10	95w3d	-	+++	-
	11	96w6d	-	+++	-
	12	96w6d	-	+++	-
	13	97w4d	**B-cell Lymphoma**	-	-
	14	98w	N.A.	N.A.	N.A.
	15	99w1d	**B-cell Lymphoma**	+	+
	16	99w4d	N.A.	N.A.	N.A.
	17	100W	**B-cell Lymphoma**	++	+++
	18	100w5d	-	-	-
	19	103w2d	-	+++	+++
	20	103w5d	**B-cell Lymphoma**	-	-
	21	103w 6d	**B-cell Lymphoma**	-	-
	22	104w	-	++	++
	23	104w5d	-	-	-
	24	105w3d	**Histiocytic sarcoma**	-	-
	25	108w6d	-	++	++
	26	114w	N.A.	N.A.	N.A.
	27	114w6d	Angiosarcoma	-	-
	28	115w3d	-	-	-
	29	117w	-	+	-
	30	120w5d	-	-	-
	31	125w1d	**B-cell Lymphoma**	-	-

NA: Not available for assessment.

S82A mice also showed extensive non-neoplastic liver and kidney disease (Fig 1B), characterized by polycystic changes affecting variable proportions of the organ parenchyma, that did not have an overall impact on survival. Mild to moderate lymphocytic infiltrates were present within the fibrous stroma surrounding cysts. Liver polycystic disease (LPD), frequently severe with near-complete effacement of healthy liver tissue, occurred in eleven of twenty-eight S82A mice, (39.3%). Microscopically, hepatic cystic structures were lined by cuboidal epithelium consistent with bile duct origin. Cystic changes were also seen in kidneys for five (17.9%) S82A mice. Microscopically, renal cysts were lined by cuboidal to attenuated epithelial cells. All mice with polycystic changes in kidneys also had polycystic changes in the liver. No cystic changes were seen in either the liver or the kidneys among control WT littermates on either gross or microscopic examination. Immunoblotting of lysates made from various organs and tissues showed that 4E-BP1 expression levels in S82A homozygous mice was comparable to WT mice formally confirming that there is no disruption of 4E-BP1 expression by the generation of the knock-in model. However, 4E-BP1 S82 phosphorylation deficiency appeared to lead to a more hyperphosphorylated 4E-BP1 profile specifically in liver tissue from S82A homozygous mice (S2 Fig).

### Sublethally irradiated S82A mice are prone to immature T-cell lymphoma with Pten-mutation

**Fig 2:**
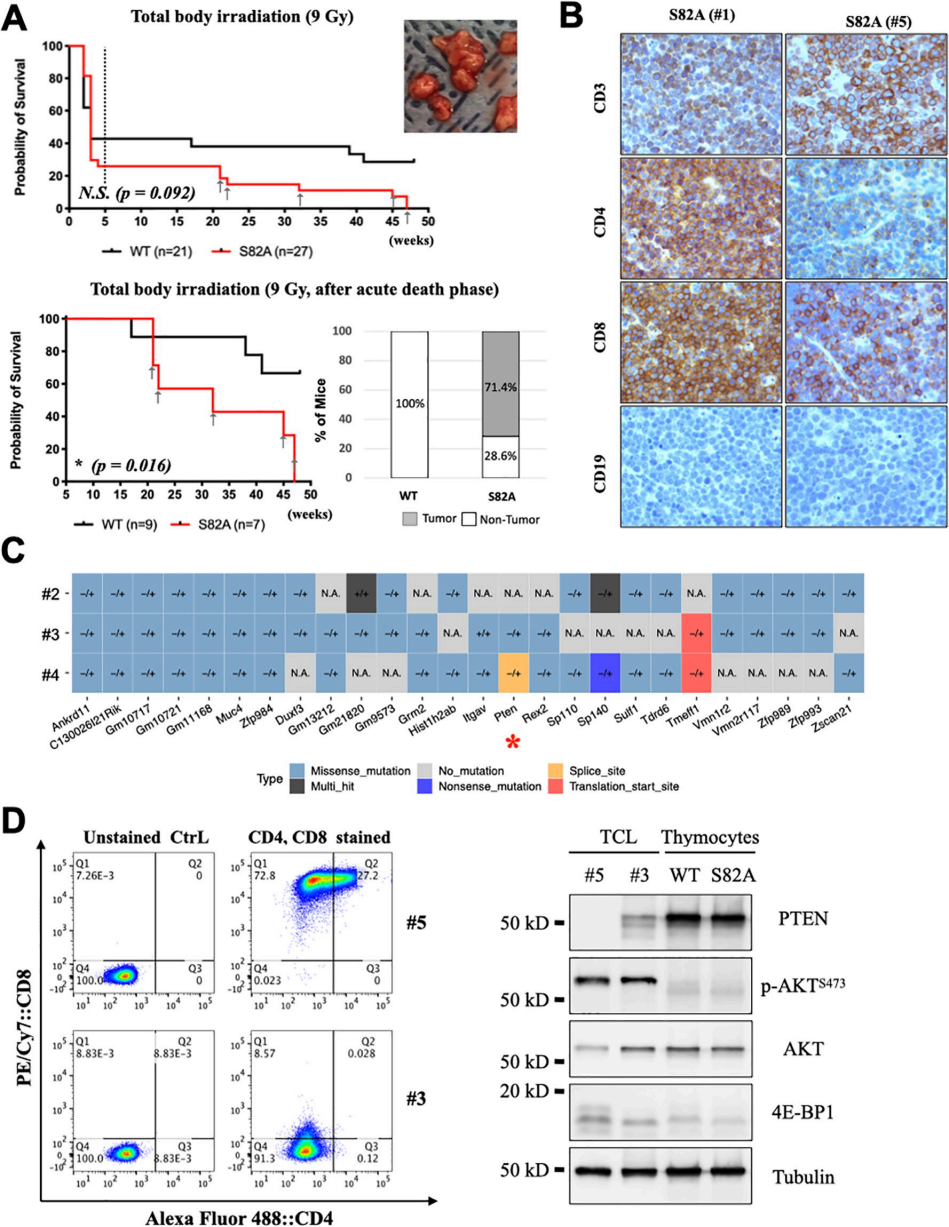
Sublethally irradiated S82A mice are prone to immature T-cell lymphoma with Pten-mutation. **(A)** Kaplan–Meier survival curve after 9 Gy total body irradiation. 11~13-week-old WT mice (n = 21, black line) and S82A mice (n = 28, red line) were subjected to total body irradiation (9 Gy). Mice with 20% weight loss or illness were euthanized for routine anatomy and histology examination. Dash line is used to indicate the end of acute death phase (5 weeks post-irradiation). Mice that died with tumors are marked with grey arrows. The picture insert (upper right corner) shows representative tumor tissues isolated from mediastinal region. Lower panel shows normalized partial Kaplan–Meier survival curve for long-term survival after irradiation. WT mice (n = 9, black line) and S82A mice (n = 7, red line) that survived the acute death phase were followed for long-term observation. Log-rank (Mantel-Cox) test was used to compare survival probability. Significance is indicated with an asterisk * (p = 0.016). Mice dying with tumors are marked with grey arrows. Column plot (right panel) summarizes the percentage of S82A and WT found with tumors. **(B)** Immunohistochemical confirmation of T-cell tumors isolated from mediastinal regions of two S82A mice (#1 and #5). Tumors were immunostained with CD3, CD4, CD8 and CD19 antibody. Brown signal indicates positive reactivity with respective antibodies. Images were captured at 40x magnification. **(C)** Common mutations identified in at least two 4E-BP1^S82A^ lymphoma tissues (#2, #3, and #4) by Oncoplots. Types of mutations are color-coded, and zygosity of mutations: heterozygous (-/+), homozygous (+/+), and not applicable (N.A.) are indicated for each mutations identified. **(D)** CD4 and CD8 staining profiles for T-cell lymphoma (TCL) cell lines (Left), #5 and #3 were established from two different tumor explants. Immunoblotting for T-cell lymphoma (TCL) cell lines (Right). Thymocytes were acquired from 4-week-old WT and S82A mice. Total cell lysates were used for immunoblotting with indicated antibodies.

To determine if neoplastic changes in S82A mice could be accelerated by stressors such as ionizing radiation (IR), eleven to thirteen-week-old S82A and WT mice were subjected to 9 Gy total body irradiation (TBI), the approximate LD_50_ for C57BL/6N mice in our irradiator facility. Twelve of twenty-one (57.1%) of WT and twenty of twenty-seven (74.1%) S82A mice died from radiation toxicity within 5 weeks post-irradiation, a difference that was not statistically significant (S3A Fig). Similarly, there was no significant difference intestinal crypt stem cell regeneration after exposure to 15 Gy TBI [23], suggesting that these cells from S82A mice were not specifically radiosensitive (S3B Fig). However, of the seven S82A mice surviving acute 9 Gy TBI radiation toxicity, five (71%) developed lymphomas at 20–47 weeks post-irradiation while none of the surviving eight irradiated WT mice developed lymphoma (Table 2 and Fig 2A). Two additional S82A mice became moribund (defined as >20% body weight loss) and were euthanized but no tumors were found at necropsy. One of these mice exhibited cystic liver disease.

**Table 2:** Long-term observation on total body irradiated WT and S82A mice.

Group	Mouse #	Time after TBI	Tumor	Cystic change in Liver	Cystic change in Kidney
WT (n = 9)	1	16w5d	-	-	-
	2	38w0d	-	-	-
	3	40w1d	N.A.	N.A.	N.A.
	4	47w4d	-	-	-
	5	47w4d	-	-	-
	6	47w4d	-	-	-
	7	47w4d	-	-	-
	8	47w4d	-	-	-
	9	47w4d	-	-	-
S82A (n = 7)	1	20w1d	**T cell lymphoma**	-	-
	2	20w1d	**Lymphoma?** *	-	-
	3	21w1	**T cell lymphoma**	-	-
	4	31w2d	**T cell lymphoma**	-	-
	5	44w1d	**T cell lymphoma**	**+**	-
	6	47w0d	-	-	-
	7	47w0d	-	**+**	-

* No-phenotyping result available.

Four tumors originating in irradiated S82A mice were examined and found to have CD3+ and CD8+ or CD4+/CD8+ (double positive, DP) T cell markers, consistent with immature T-cell lymphomas (Fig 2B). The fifth S82A tumor was necrotic and could not be immunophenotyped. Among mice surviving 9 Gy TBI, S82A mice had significantly reduced long-term survival (after recovery from acute effects) compared to WT mice (Log-rank test, p = *0*.*016*) (Fig 2A). The T cell origin of lymphomas developing in irradiated mice contrasts with the B cell lymphomas developing in non-irradiated mice.

To determine if hematopoietic stem/progenitor cell (HSPC) populations differ between S82A and WT mice, bone marrow HSPC were harvested but no significant differences between LSK (Lin^-^/Sca-1^+^/c-Kit^+^) or myeloid progenitor LK (Lin^-^/Sca-1^-^/c-Kit^+^) cell populations were seen (S4A Fig). Splenocytes and thymocytes harvested from 6-week-old WT and S82A mice also showed similar populations of CD4+, CD8+, DP and double-negative (DN) T cells by flow cytometry (S4B Fig). No significant differences in immature T cell populations were found between S82A and WT mice (S4C Fig).

To identify driver mutations in S82A lymphomas, whole genome sequencing was performed on 3 lymphoma and 1 non-irradiated S82A brain tissue and 26 lymphoma-related mutations were identified (Fig 2C). Seven gene mutations (*Ankrd11*, *C130026I21 Rik*, *Gm10717*, *Gm10721*, *Gm11168*, *Muc4*, *and Zfp984)* were hetero-/hemi-zygous in all three lymphomas and one gene (*Itgav)* had homozygous mutations in one lymphoma. Two lymphomas contained mutations in *Pten*, a common lymphoma driver gene: one contained a missense mutation, and another contained a splice site mutation. Cell lines were established from two lymphomas arising in the irradiated S82A mice (Fig 2D and Table 2) and had reduced or absent PTEN expression compared to control thymocytes isolated from S82A and WT mice (Fig 2D), suggesting irradiated S82A mice are more prone to PTEN mutation.

## Discussion

Since cell culture models have not revealed a physiological function for phosphorylation of the 4E-BP1 S82 residue [18,21], we sought to examine its role in mice in which the S82 residue alone is mutated to a non-phosphorylated alanine. These unstressed, homozygous mice were generally healthy, fertile and had similar lifespans to wild-type littermate control mice, consistent with all other 4E-BP1 regulatory sites (e.g., T37, T46, T70, and S65) being intact and regulated by mTOR and CDK1 in a similar manner to the wild-type protein. The S82A knock-in mutation resulted in tissue-specific pathologies, particularly B and T cell lymphomagenesis and liver/kidney polycystic disease that was only evident at necropsy. These effects were markedly accelerated after recovery from the stress of TBI, leading to premature mortality in the S82A homozygotes.

It has been shown that intra-thymic T cells can be relatively resistant and auto-reconstitute after irradiation unlike T cells from other hematopoietic and lymphoid organs [24]. This may potentially explain the T cell lymphoma phenotype in S82A homozygous mice. Previous studies have reported Trp53(p53) knockout mice to spontaneously develop immature T cell lymphomas in their first six months of life with all tumour samples having lost or reduced Pten [25]. We found similar Pten results in 4e-bp1^S82A^ T cell lymphoma samples (Fig 2D). The critical role of Pten in preventing T cell lymphomagenesis has been well documented and studied [26]. However, we did not find apparent p53 mutation or deletion in 4e-bp1^S82A^ tumour samples. Meanwhile, we found intact Pten expression and normal T cell development in young 4e-bp1^S82A^ mice, which suggests that additional stress stimuli are essential for 4e-bp1^S82A^ mice lymphomagenesis. 4e-bp1^S82A^ mice may be better able to retain and allow expansion of mutated cells resulting from aging or sublethal irradiation.

Polycystic kidney disease (PKD) has been described as a “neoplasia in disguise” [27] that may be related to perturbed mTOR signaling [28]. PKD is the most common hereditary cause of kidney failure and is commonly associated with liver cystic liver changes [29]. Holditch and colleagues found elevated 4E-BP1 hyperphosphorylation in human PKD human tissues and kidneys from PKD-prone mice [30]. PKD disease severity in PKD mice, however, was unexpectedly increased after viral vector transduction of mTOR-resistant 4E-BP1^F113A^ consistent with 4E-BP1 dysregulation, rather than inhibition, contributing to PKD. Recent studies also point towards CDK1 dysfunction predisposing to cystic organ disease [31], which is supported by our study. Since cystic disease was mainly evident in aging S82A mice, our data suggests that 4E-BP1 S82 phosphorylation by CDK1 may be critical for tissue renewal of differentiated cell populations rather than progenitor cell populations involved in organogenesis.

Even though we did not find clear mechanisms to account for the phenotype observed in the 4E-BP1^S82A^ mouse, this study provides some intriguing data suggesting that 4E-BP1 S82 phosphorylation is beneficial for overcoming some cellular stress conditions such as aging and irradiation. Several important caveats should be considered in interpreting our data. It is possible that 4E-BP1^S82^ phosphorylation is critical to a tissue or specific cell type more pertinent to the phenotype that we did not sample in our studies (e.g., liver or kidney cells; B cell; other T cell populations). Although markers such as CD34, cytoplasmic CD3 or deoxynucleotidyl transferase [32] may help define the origin and identity of the immature T-cell lymphomas in our study, we did not perform this immuno-profiling on the lymphoma samples. We did not track lymphomagenic capacity after irradiation, and perhaps in the T cell population this may have revealed differences in regeneration between the S82A and WT mice. Since the observed phenotype in aged S82A mice develops very late, mating this S82A mouse strain with an aging mouse model strain might reproduce the observed phenotype at an earlier age.

Nevertheless, despite these caveats, this mouse strain has utility in separating the effects of CDK1 from mTOR on 4E-BP1 function and provides a new model for polycystic organ disease that is open to therapeutic interventions.

## Materials and methods

### Ethics statement

Mice breeding and long-term monitoring experiment was approved by the Institutional Animal Care and Use Committee (IACUC) guidelines and were approved by the Animal Ethics Committee at University of Pittsburgh (IACUC breeding protocol #18011983, IACUC experimental protocol #18012088, and TBI protocols #18022000 and #18073023). Per protocol, mice were sacrificed by using 100% CO_2_ followed by cervical dislocation if mice developed tumors greater than 1.8 cm in diameter, or if the mice showed any signs of persistent morbidity such as loss in weight greater than 20%, lethargy, unwillingness to ambulate, hunched posturing and ruffled fur. No invasive procedures likely to produce moderate to severe pain were performed; 3–5% isoflurane inhalant induction and maintenance was performed for genotyping studies. All efforts were made to minimize possible pain and suffering for mice during irradiation, monitoring, and euthanasia.

### Generation of 4E-BP1^S82A^ (S82A) knock-in mouse

4E-BP1 Ser(S)83 phosphorylation is evolutionally conserved in vertebrates and the human 4E-BP1 S83 residue corresponds to mouse 4E-BP1 S82 (Fig 1A). To study the physiological function of 4E-BP1 S83 phosphorylation, knock-in mice were generated commercially (Gen-O-Way) that express phosphorylation-defective 4E-BP1 containing S82_AGC_-to-Ala(A)82_GCT_ (S82A) mutation. Briefly, 4E-BP1^S82A^ allele was introduced to the mouse embryonic stem (ES) cell genome through homologous recombination. The successfully targeted ES cells were verified by Southern hybridization (S1B Fig) and injected into blastocysts to develop the chimeric mice. The chimeric male mice were mated with C57BL/6N Cre female mice to excise loxP-Neo cassette. Mice harboring germline-transmitted 4E-BP1^S82A^ allele were selected as heterozygous founders. S82A homozygous mice were established by heterozygous breeding. Homozygous and heterozygous S82A mice were fertile and not obviously different from WT mice in Mendelian sex ratios, perinatal mortality or weight gain.

### Mouse experiments

For long-term observation, mouse body weight was monitored every week. Mice with 20% body weight loss or compromised body conditions were euthanized. Tissues were harvested, formalin-fixed, and embedded in paraffin blocks (FFPE). For irradiation experiments, 11~13-week-old mice were subjected to total body irradiation at 9 Gy. Mice survived from acute irradiation syndrome were monitored every week. Euthanasia criteria are the same as in long-term observation mice. Survival curves were made with Prism GraphPad.

### Immunohistochemistry (IHC) and pathologic analysis

Mouse tissues were fixed in 10% neutral buffered formalin (Sigma) and embedded in paraffin, then sectioned (5 μm) onto glass slides. Slides were deparaffinized and rehydrated using a standard histology protocol. Antigen retrieval was performed using 10 mM sodium citrate pH 6.0 buffer and a Decloaking chamber at 120°C. The slides were stained using an Autostainer Plus (Dako) with TBST rinse buffer (Dako). The following antibodies were used for IHC: anti-CD4 (Cell Signaling, D7D2Z, 1:100), anti-CD8 (Cell Signaling, D4W2Z, 1:400), anti-CD3 (Biocare Medical, 1:100), and anti-CD19 (Cell Signaling, D4V4B, 1:800). Detection consisted of Rabbit Boost (Cell Signaling) HRP polymer used with 3,3’-diaminobenzidine (DAB) (Dako). Slides were finally counterstained with hematoxylin (Dako). Images were captured with an Olympus AX70 microscope using the Q-Capture Pro7 Program. H&E stained sections and immunohistochemistry of lymphoid malignancies and polycystic changes were evaluated by two board-certified pathologists including a veterinary pathologist (LHR).

### T-cell lymphoma (TCL) cell lines

Tumor explants were minced and cultured in RPMI-1640 (Corning Cellgro) supplemented with 10% FBS, non-essential amino acid and 100 U/ml Penicillin/Streptomycin.

### Immunoblotting

Cells were lysed in RIPA buffer (50 mM Tris·HCl [pH 7.4], 150 mM NaCl, 0.5% Triton X-100, 2 mM Na3VO4, 2 mM NaF) supplemented with protease inhibitors (Roche). Lysates were boiled in SDS loading buffer and subjected to 12% SDS-PAGE and transferred to nitrocellulose membranes. Membranes were blocked with 5% milk and incubated with primary antibodies in 5% BSA overnight at 4°C. After washing, blots were subsequently incubated with IRDye-labeled anti-rabbit or anti-mouse secondary antibodies (LI-COR Biosciences) and analyzed by ChemiDoc imaging system (Bio-Rad). The following primary antibodies were used in this study: anti-4E-BP1 (Cell Signaling, 53H11), anti-PTEN (Cell Signaling, 138G6), anti-phospho-Akt^S473^ (Cell Signaling, D9E), anti-Akt (Cell Signaling, C67E7), anti-alpha-Tubulin (DSHB, 12G10), and anti-phospho-4E-BP1^S83^ (Millipore, ABE2889).

### Thymocytes and splenocytes isolation

Spleen and thymus from 4~8-week-old mice were harvested in R10 media (RPMI-1640 supplemented with 10% FBS, non-essential amino acid, sodium pyruvate, 100 U/ml Penicillin/Streptomycin, 20 mM HEPES and 50 μM β-ME) on ice. Collected tissues were ground between frosted glass slides and filtered through 70 μm filter. Pelleted cells were resuspended in 1 ml RBC lysis buffer (0.15 M NH_4_Cl, 10 mM NaHCO_3_, 0.1 mM EDTA, pH 8.0) for 30–40 seconds and diluted with 4 ml serum free media immediately after lysis. Cells were washed with R10 media and filtered through 70 μm filter for downstream experiment.

### Flow cytometry

For cell surface proteins, cells were stained with fluorescence-conjugated antibodies in FACS buffer (2% FBS in PBS) on ice for 30 min, and stained with eFluor 780 viability dye (Thermo Fisher, #65-0865-14, 1:3000) or Zombie Violet fixable viability dye (Biolegend, #423113, 1:1000) in PBS on ice for 15 min, followed by fixation with IC fixation buffer (Thermo Fisher, #88-8824-00, 1:1 with FACS buffer) for 15 min. The following antibodies were used: anti-CD8 PE-Cy7 (Biolegend, #100721, 1:500), anti-CD4 Alexa-Fluor-488 (Biolegend, #100425, 1:500), anti-CD4 APC-Cy7 (Biolegend, #100413, 1:500), anti-CD44 Alexa-Fluor-647 (Biolegend, #103017, 1:500), and anti-CD25 Pacific Blue (Biolegend, #102021, 1:500). Data was acquired by using LSR Fortessa (4 laser) analyzer (Becton Dickinson) and analyzed with FlowJo software.

### Whole genome sequencing and mutation analysis

Paired end Illumina sequencing reads were checked for quality and mapped to the mouse genome (mm10) using the DNA Nexus platform’s pipeline. Mutect (v1.1.7) and Varscan2 (v2.4.2) was used for identifying somatic variants. Variants were annotated using Vcf2maf (v1.6.16) and Variant Effect Predictor (VEP v95) to produce mutation annotation format (MAF) files. A union of annotated variants from Mutect and Varscan2 was used for further analysis. Oncoplots were generated using maftools (v2.2.0). The zygosity is estimated by the variant calling tools.

## Supporting information

S1 Fig 4e-bp1^S82A^ mice construction and verification. **(A)** 4E-BP1 Ser83 phosphorylation is conserved among higher order organisms. Protein sequences were aligned using Clustal Omega method. Conserved amino acids are highlighted in yellow. The Cre-*loxP* cloning strategy for 4E-BP1^S82A^ knock-in mice is illustrated (right). **(B)** Southern hybridization for targeted ES cells after DTA and G418 selection. Genomic DNA digested with *Spe*I and AvrII was detected by probe A. Genomic DNA digested with EcoN1 was detected by probe B. The detection of 5.1 kbp fragment and 7.2 kb fragment both indicate successful recombination. **(C)** Genome typing for 4e-bp1^S82A^ point mutation knock-in mice. PCR with primers flanking *loxP* site generated 229 bp band for 4e-bp1^S82A^ mice (#2) and 162 bp band for 4e-bp1^WT^ mice (#3, 4, 6). Two bands can be amplified from heterozygous littermate (#1, 5). **(D)** Immunoblotting for activated 4e-bp1^WT^ and 4e-bp1^S82A^ CD8+ T cells. STLC treatment (5 μM; 6 h) was used to arrest cells in mitosis, which enriches 4E-BP1 Ser82 phosphorylation in 4e-bp1^WT^ cells. Total cell lysates were used for immunoblotting with indicated antibodies.
(TIF)

S2 Fig 4E-BP1 expression level in S82A homozygous mice is comparable to WT counterpart. The indicated tissues or organs were collected from 7~9-week-old 4E-BP1^WT^ and 4E-BP1^S82A^ mice (three for each group), and lysed in RIPA buffer. Total cell lysates were used for immunoblotting with labeled antibodies.
(TIF)

S3 Fig Supplemental TBI radiation data and crypt microcolony assay. **(A)** Kaplan–Meier survival curve for mice in acute death phase related to Fig 2A. **(B)** Crypt microcolony assay to quantify stem cell survival by counting regenerated crypts in H&E-stained cross sections 96 hours after 15 Gy TBI. Representative images of the small intestine of WT and S82A are shown. Bar = 100 μm. Arrow indicates a characteristic single, regenerated crypt. Regenerated crypts were quantitated (2 mice/group, 6–8 full cross-sections per mouse). ns, not significant, P = 0.06, Student’s t-test, two-tailed. The data are reported as means ± SEM.
(TIF)

S4 Fig S82A homozygous mice have normal T-cell hematopoiesis. **(A)** Flow cytometry profiles of HSPC subsets from bone marrow. Bone marrow cells were isolated from 7~9-week-old 4E-BP1^WT^ and 4E-BP1^S82A^ mice. After staining with eFluor780 viability dye, cells were incubated with lineage cocktail (c-Kit and Sca-1) for surface staining. No difference was observed between S82A and WT cells in the percentage of Lin^-^/c-Kit^+^ (LK), Lin^-^/Sca-1^+^ (LS) and Lin^-^/Sca-1^+^/c-Kit^+^ (LSK) cells. Three independent experiments were performed. Error bars represent SD. **(B)** Flow cytometry profiles of T cell subsets in thymus and spleen. Thymocytes and splenocytes from 6-week-old S82A and WT mice were stained with fluorescence dye conjugated CD4 and CD8 antibodies. The ratio of gated CD4, CD8 double positive (DP), CD4 or CD8 single positive (SP), and CD4, CD8 double negative (DN) population are indicated. Data shown is a representative result of multiple independent experiments. **(C)** Flow cytometry profiles of CD4, CD8 DN T-cell subsets in thymus. Thymocytes from 6-week-old WT and S82A mice were stained with fluorescence dye conjugated CD4, CD8, CD25, and CD44 antibodies. CD25-CD44 plots were gated on the CD4, CD8 double negative (DN) population. The ratio of different stages of DN T-cells (DN1-4) are indicated. The data is the representative result of multiple independent experiments.
(TIF)

S1 File Supplemental materials and methods. (DOCX)

S2 File Raw figures for reference. (PDF)
