# Supplementary figures and images for: Mitotic CDK1 and 4E-BP1 I: Loss of 4E-BP1 serine 82 phosphorylation promotes proliferative polycystic disease and lymphoma in aged or sublethally irradiated mice
Source: PLoS One. 2023 May 5;18(5):e0282722. doi: 10.1371/journal.pone.0282722 (PMC10162543; doi:10.1371/journal.pone.0282722)

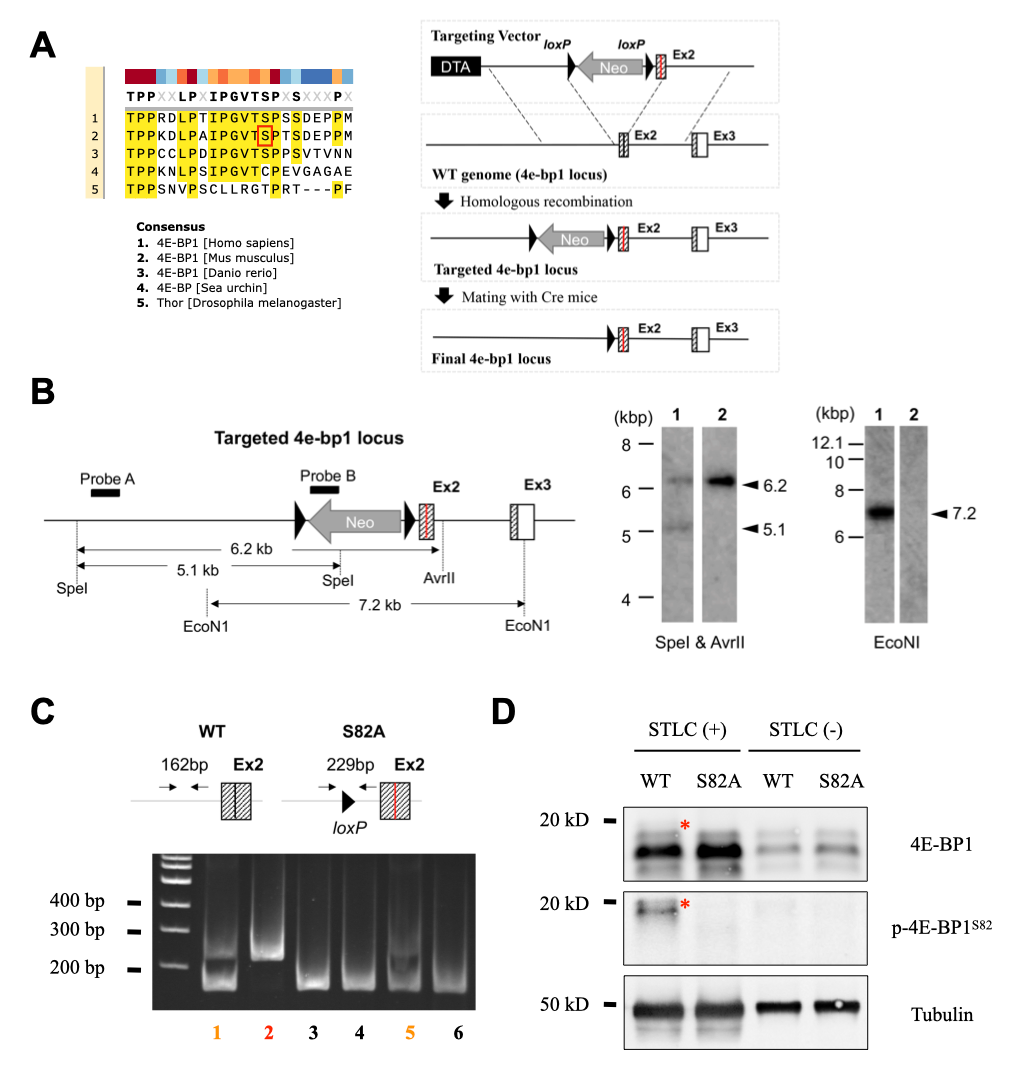

Supplement: S1 Fig — (A) 4E-BP1 Ser83 phosphorylation is conserved among higher order organisms. Protein sequences were aligned using Clustal Omega method. Conserved amino acids are highlighted in yellow. The Cre-loxP cloning strategy for 4E-BP1S82A knock-in mice is illustrated (right). (B) Southern hybridization for targeted ES cells after DTA and G418 selection. Genomic DNA digested with SpeI and AvrII was detected by probe A. Genomic DNA digested with EcoN1 was detected by probe B. The detection of 5.1 kbp fragment and 7.2 kb fragment both indicate successful recombination. (C) Genome typing for 4e-bp1S82A point mutation knock-in mice. PCR with primers flanking loxP site generated 229 bp band for 4e-bp1S82A mice (#2) and 162 bp band for 4e-bp1WT mice (#3, 4, 6). Two bands can be amplified from heterozygous littermate (#1, 5). (D) Immunoblotting for activated 4e-bp1WT and 4e-bp1S82A CD8+ T cells. STLC treatment (5 μM; 6 h) was used to arrest cells in mitosis, which enriches 4E-BP1 Ser82 phosphorylation in 4e-bp1WT cells. Total cell lysates were used for immunoblotting with indicated antibodies. (TIF) [file pone.0282722.s001.tif]

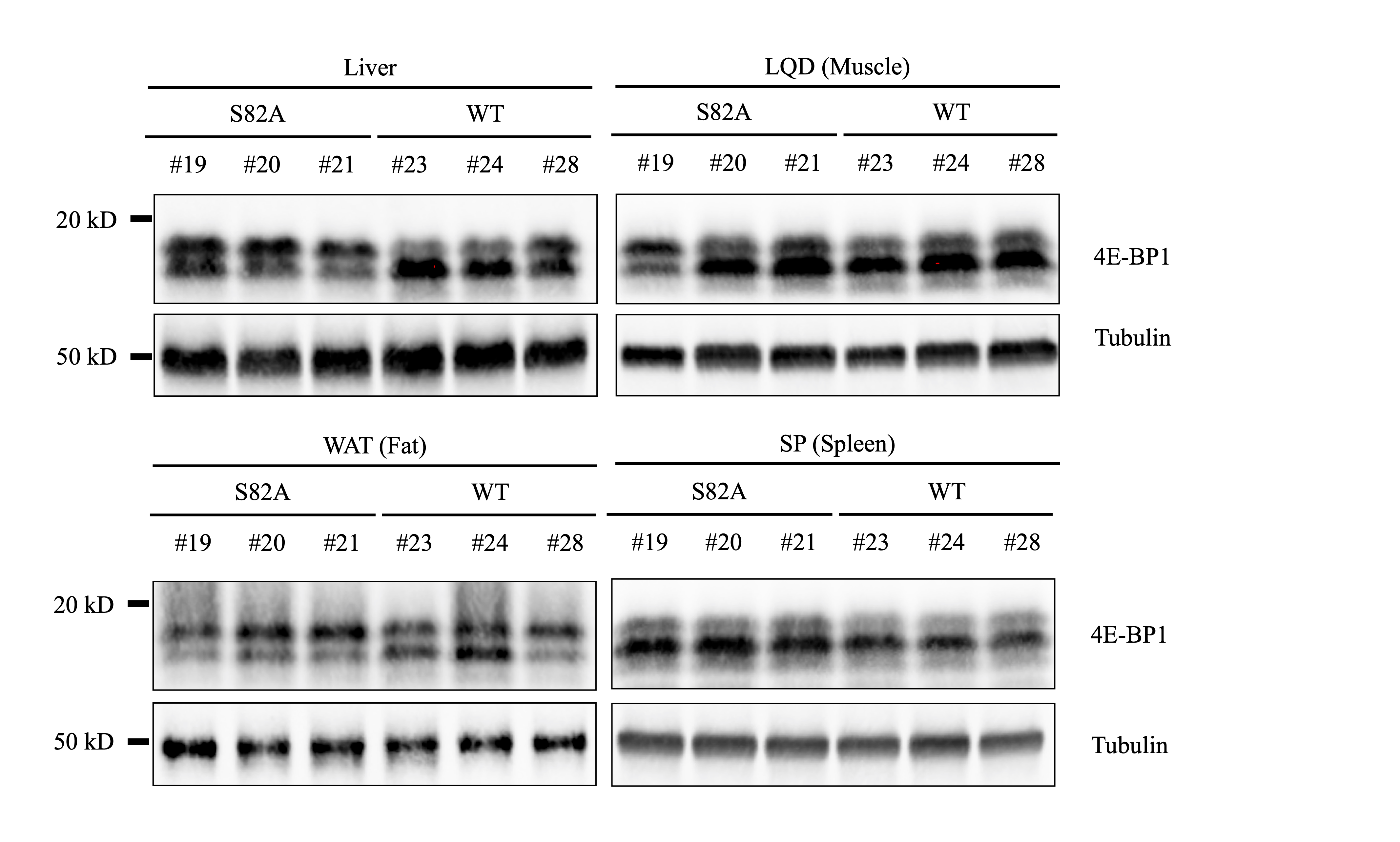

Supplement: S2 Fig — The indicated tissues or organs were collected from 7~9-week-old 4E-BP1WT and 4E-BP1S82A mice (three for each group), and lysed in RIPA buffer. Total cell lysates were used for immunoblotting with labeled antibodies. (TIF) [file pone.0282722.s002.tif]

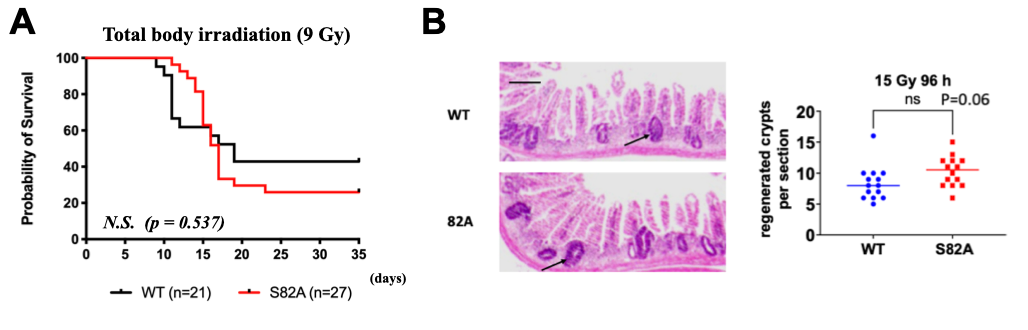

Supplement: S3 Fig — (A) Kaplan–Meier survival curve for mice in acute death phase related to Fig 2A. (B) Crypt microcolony assay to quantify stem cell survival by counting regenerated crypts in H&E-stained cross sections 96 hours after 15 Gy TBI. Representative images of the small intestine of WT and S82A are shown. Bar = 100 μm. Arrow indicates a characteristic single, regenerated crypt. Regenerated crypts were quantitated (2 mice/group, 6–8 full cross-sections per mouse). ns, not significant, P = 0.06, Student’s t-test, two-tailed. The data are reported as means ± SEM. (TIF) [file pone.0282722.s003.tif]

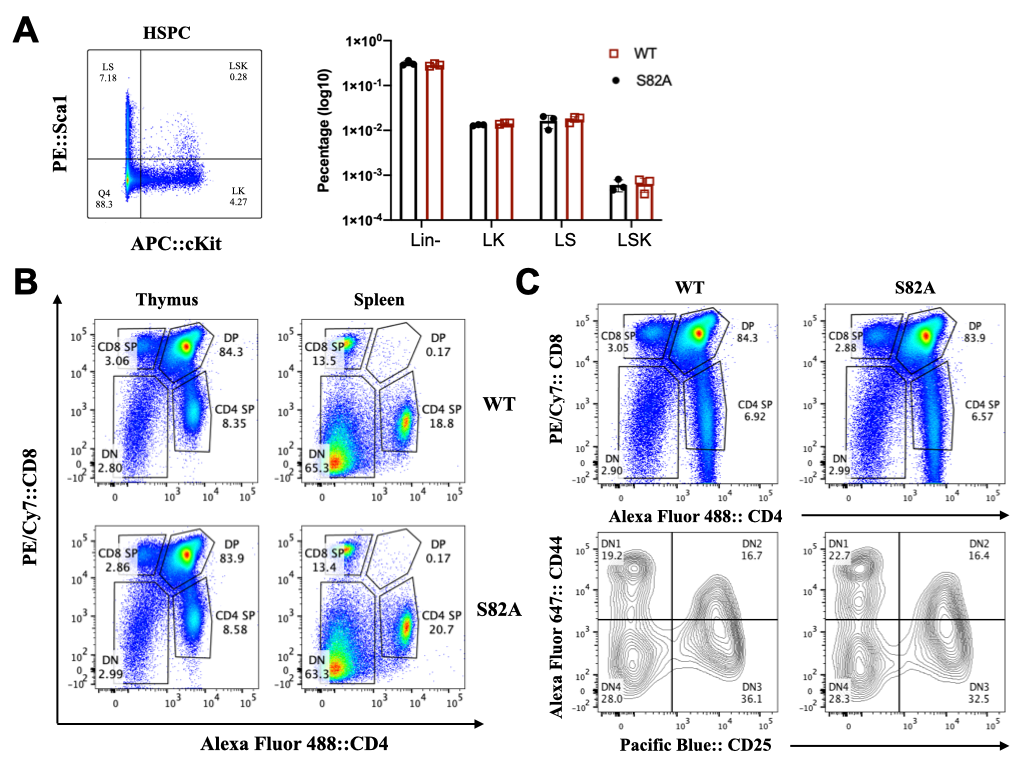

Supplement: S4 Fig — (A) Flow cytometry profiles of HSPC subsets from bone marrow. Bone marrow cells were isolated from 7~9-week-old 4E-BP1WT and 4E-BP1S82A mice. After staining with eFluor780 viability dye, cells were incubated with lineage cocktail (c-Kit and Sca-1) for surface staining. No difference was observed between S82A and WT cells in the percentage of Lin-/c-Kit+ (LK), Lin-/Sca-1+ (LS) and Lin-/Sca-1+/c-Kit+ (LSK) cells. Three independent experiments were performed. Error bars represent SD. (B) Flow cytometry profiles of T cell subsets in thymus and spleen. Thymocytes and splenocytes from 6-week-old S82A and WT mice were stained with fluorescence dye conjugated CD4 and CD8 antibodies. The ratio of gated CD4, CD8 double positive (DP), CD4 or CD8 single positive (SP), and CD4, CD8 double negative (DN) population are indicated. Data shown is a representative result of multiple independent experiments. (C) Flow cytometry profiles of CD4, CD8 DN T-cell subsets in thymus. Thymocytes from 6-week-old WT and S82A mice were stained with fluorescence dye conjugated CD4, CD8, CD25, and CD44 antibodies. CD25-CD44 plots were gated on the CD4, CD8 double negative (DN) population. The ratio of different stages of DN T-cells (DN1-4) are indicated. The data is the representative result of multiple independent experiments. (TIF) [file pone.0282722.s004.tif]

**Fig S1C**

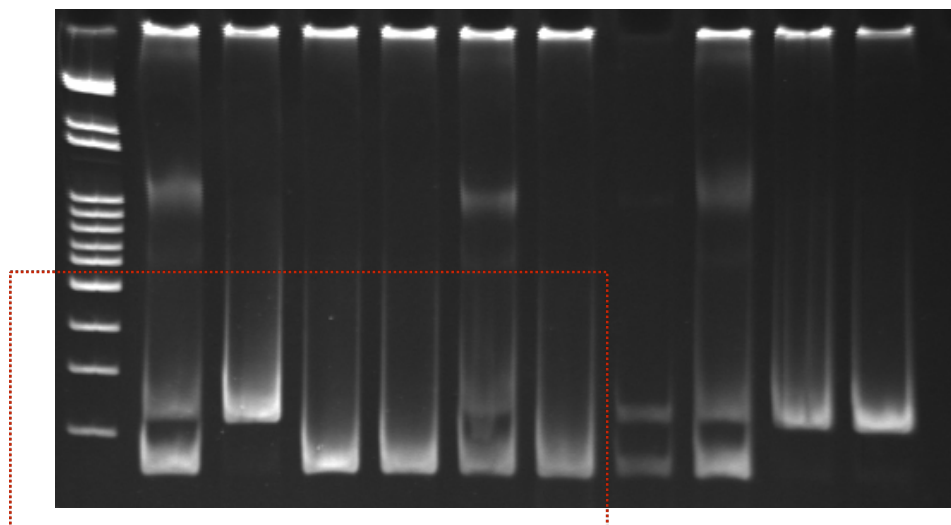

**Fig S1D**

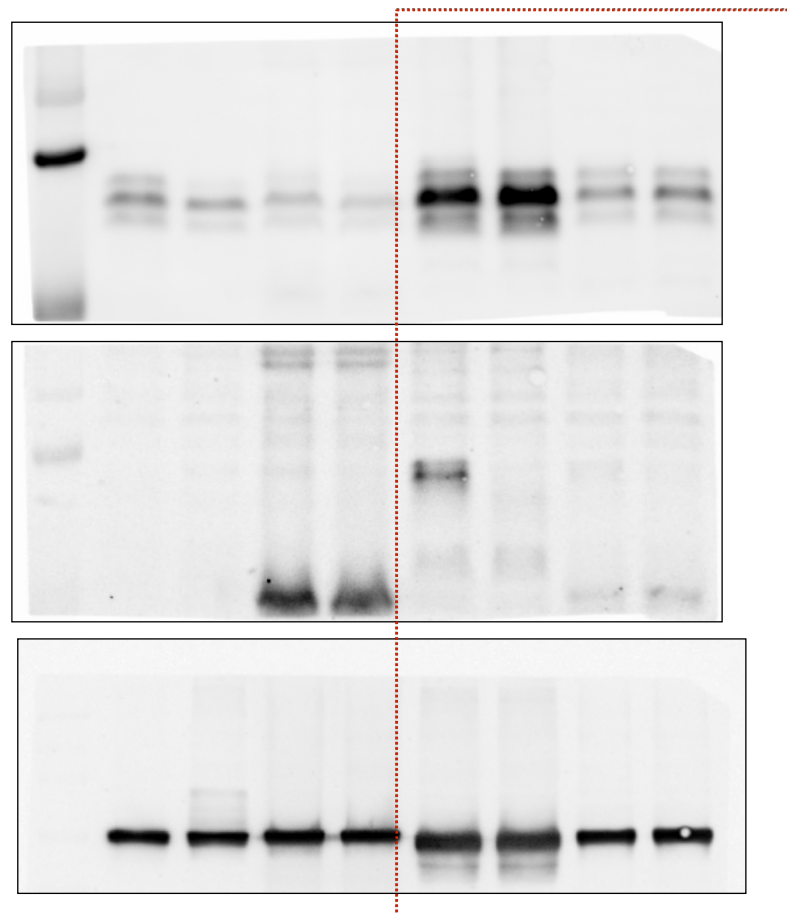

**Fig S2**

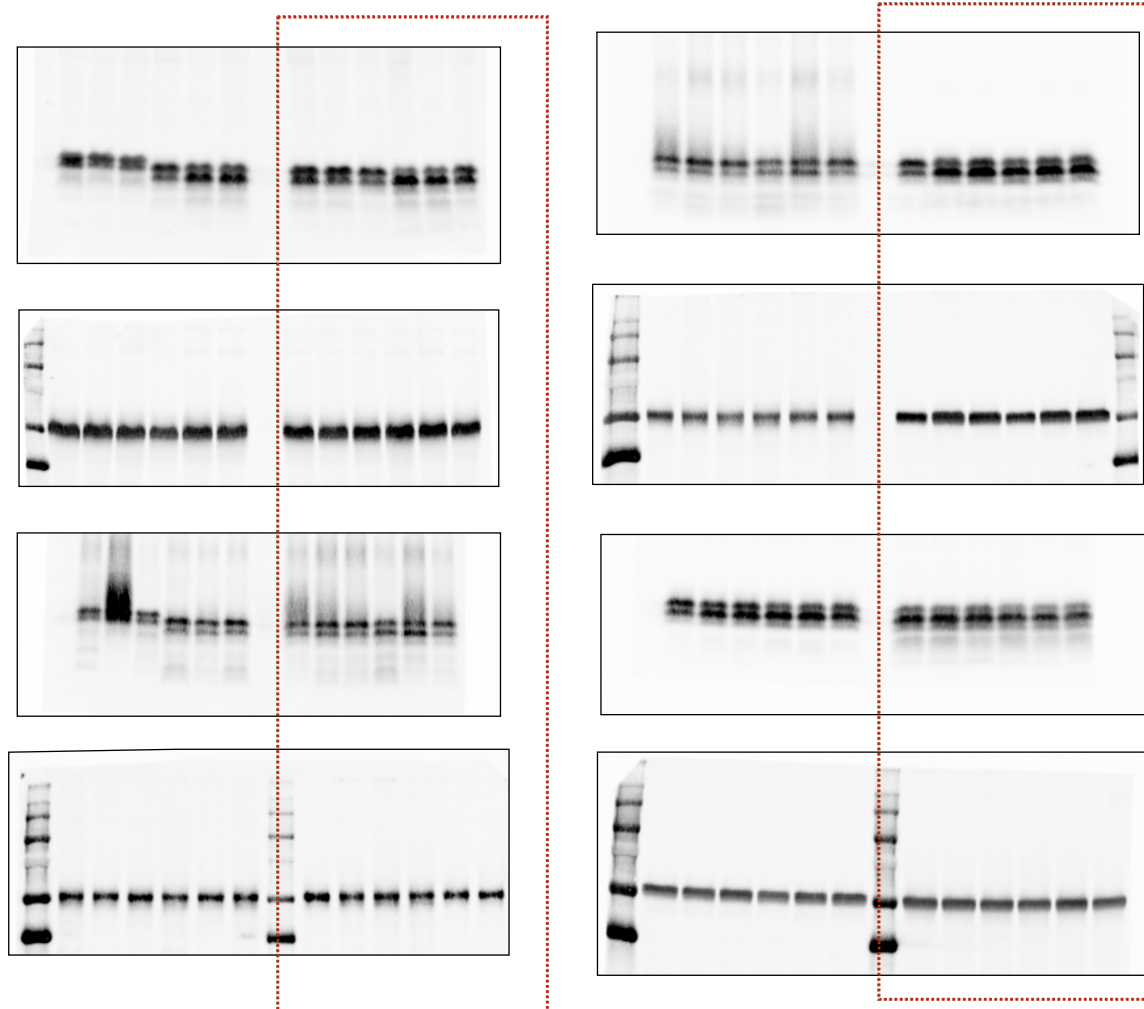

**Fig 2D**

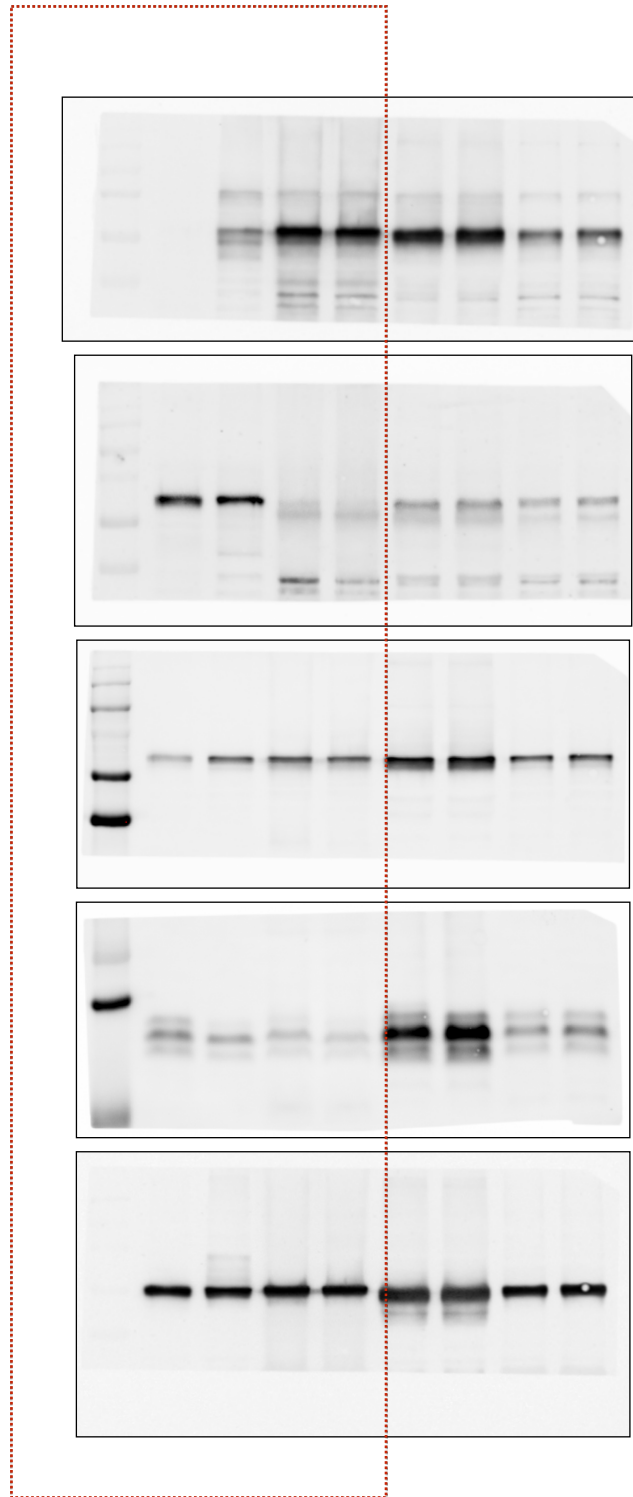

Supplement: S2 File — (PDF) [file pone.0282722.s006.pdf]
