# Supplementary material for: Mitotic CDK1 and 4E-BP1 I: Loss of 4E-BP1 serine 82 phosphorylation promotes proliferative polycystic disease and lymphoma in aged or sublethally irradiated mice
Source: PLoS One. 2023 May 5;18(5):e0282722. doi: 10.1371/journal.pone.0282722 (PMC10162543; doi:10.1371/journal.pone.0282722)
Supplement: S1 File — (DOCX) [file pone.0282722.s005.docx]

**Supplemental Materials and Methods**

**Genotyping.** Ear snips were boiled in 100 μL 50 mM NaOH at 95°C for 15 min, and then neutralized by the addition of 5 μL Tris buffer (1.0 M, pH 8.0). Genomic DNA was extracted from the supernatant. PCR primers were listed in **Table S1**. The annealing temperature and cycles for PCR reaction was 58°C and 30. Representative genotyping results are shown in **Fig S1C**. The expected product sizes are ~230 bp for homozygous 4e-bp1^S82A^ and ~160 bp for 4e-bp1^WT^.

Table S1: Primers used in this study

| CM Log | Name | Sequence | Notes |
| --- | --- | --- | --- |
| # 6819 | 033cof-PAM2 | CCTATGCTATAGTCCCTCAACCTCTCATCC | Genotyping |
| # 6818 | 034cof-PAM2 | GGACCCACTGTCTTTCTGTTGTGTAGATG | Genotyping |

**Crypt microcolony assay.** Crypt microcolony assay is described previously (*1, 2*). In brief, the proximal jejunum was removed immediately after sacrifice, rinsed with ice-cold saline, and cut into 6-8 one cm sections that were bundled together with 3M Micropore tape. Tissue bundles were fixed overnight in 10% neutral-buffered formalin (Sigma) prior to paraffin embedding. Tissue sections (5 µm) were deparaffinized and rehydrated through graded ethanol, then H&E stained. Surviving crypts were defined as containing 5 or more adjacent chromophilic non-Paneth cells, at least one Paneth cell and a lumen.

**CD8+ T cell *in vitro* stimulation and expansion.** Purified CD8+ naïve T cells were resuspended in the R10 medium containing 2 μg/ml anti-CD28 (Becton Dickinson, #553294) and 10 ng/ml IL-2 (Biolegend, #575404), and cultured in the plate that was pre-treated with 10 μg/mL anti-CD3ε (Biolegend, #100331). Cells were activated in the 37 ºC incubator for at least 24 hours. After 24 hours stimulation, cells were washed and resuspended in R10 media containing 10 ng/ml IL-2 and cultured in the plate without anti-CD3ε coating. Equal volume fresh R10 media with IL-2 were added to the plate or flask every 24 hours.

**REFERENCES FOR SUPPLEMENT**

1. W. Qiu *et al.*, PUMA regulates intestinal progenitor cell radiosensitivity and gastrointestinal syndrome. *Cell Stem Cell* **2**, 576-583 (2008).

2. L. Wei *et al.*, Inhibition of CDK4/6 protects against radiation-induced intestinal injury in mice. *J Clin Invest* **126**, 4076-4087 (2016).
